# The “Correction” of Professor Dr. G. Jaeger from the Hahnemannian Standpoint

**Published:** 1893-02

**Authors:** B. Fincke

**Affiliations:** Brooklyn, N. Y.


### THE "CORRECTION" OF PROF. DR. G. JAEGER, FROM THE HAHNEMANNIAN STANDPOINT.

In the *Allg. hom. Zeitung*, vol. 125, p. 157.

B. Fincke, M. D., Brooklyn, N. Y.

The premise "a medicine is *matter* and *dead* matter at that," cannot be admitted from the Hahnemannian standpoint. A medicine is a force which acts upon the living body, sickening or healing. The circumstance that this force is bound up in a substance which, in combination with another substance, shows physical and chemical action, does not render matter a medicine. Just as the physical and chemical force is bound up in the substance which serves as a vehicle for it, just so the medicinal force is bound up in matter. Therefore, in our case, not a theory of the action of matter is in question, but a theory of the force which, being bound up in matter, becomes liberated by potentiation and transferred upon inert substances as further vehicles in order to serve as medicine to the healing art. Consequently a theory of the action of the medicinal force must be conceived to be dynamical. The idea that it is the matter which occasions the physical and chemical phenomena is an emanation of the doctrine of Materialism which makes matter appear dead at the very time when in these phenomena it shows itself to be very much alive. The dynamism lies at the bottom of these phenomena just as well as of those observed of medicines in living beings. This conception suffers no change by conceding that also "non-materialistic processes take place in living beings," for with this admittance the whole correction is demolished. For, if it is proved that medicines are non-materialistic potencies, where is, then, the explanation of the theory according to materialistic conception? Indeed one thing is "completely uncontended that nobody ever thought of potentiating the spirit by succussion with spirit of wine, but that has always been done with material substances." From this, however, it does not follow at all that the changes which matter undergoes are to be explained materialistically. These changes relate entirely to the problem of liberating the medicinal force from its union with the substance, by transferring it from this upon other and inert vehicles. The late neuro-analytical experiments prove, beyond a doubt, that the changes of matter which take place in the process of potentiation are not essential for potentiation itself. If the theory is supported materialistically by the molecular theory, it, at the same time, depends dynamically upon the "motor-energy of matter" spoken of in Dr. Jaeger's explanation given in his late work. Where is to be found in a one-thousandth centesimal attenuation—better potency because it is not an attenuation—the originally applied substance which, in its highest attenuation at best does not reach beyond the twelfth centesimal potency? Crookes' radiant energy does not go beyond the third centesimal, and the computation of atoms by Thompson likewise stays far behind even a twelfth centesimal potency. Furthermore, the molecular theory in its premises contradicts the laws of motion and, already for this reason, cannot furnish an explanation for potentiation.

No! We do not shake the spirit with spirit of wine as those do who, too much, enjoy the spirituous beverage, but we do shake the substance with alcohol in order to draw from it and transfer upon the inert vehicles used in potentiation that which is able to change the state of the living being into health or disease. Therefore, only the standpoint is concerned from which potentiation, being placed beyond doubt by practical experience and lately by Jaeger's Neural Analysis, is to be considered and explained. The concluding sentence in the correction is, therefore, quite correct: that "an explanation of the changes which matter undergoes in potentiation cannot by any means be otherwise than materialistic. For if it is not, then it is nothing," viz., the explanation. And such is, indeed, the case, after what has been said before. Here we have nothing to do with the spirit, to which we appoint a higher place than to a simple medicinal force, but with the medicinal force which by Nature has been assigned to matter and transferable upon other matter; this, and this alone, comes here in question. The neuro-analytician who proves the positive action of even only a one-thousandth centesimal potency (the practical experience already goes into the millions), must search for a deeper foundation of an explanation of potentiation than the almighty property of matter. Potentiation teaches us that no force can exist without matter, but it also teaches that this force can be transferred from one substance as its vehicle to another substance as vehicle, and this is in perfect accordance with the teaching of physics as the doctrine of force and matter. As the motor-force is transferred from the burning matter upon the heated water and from this upon the engine in the natural potentiation of generation of steam, so also the medicine-force is on the homœopathic method by distribution to large masses of inert vehicle transferred upon these in order now to serve as remedy. The Mechanical Theory is already accepted by science at large, why, then, should it not find its application in the healing science and art?

Honor and thanks, however, to the scientific pioneer, Professor Dr. G. Jaeger, who, though not a homœopathic physician himself, yet must be acknowledged with joy as one of our own, since, with characteristic intrepidity, leaning upon his new neuro-analytical proofs by numbers, he has supported the Hahnemannian doctrine by the following expressions:

1. "My neuro-analytical discovery is nothing else but what Hahnemann has taught."

2. "The only right way after all is that one takes the matter in hand himself and tries it, and that upon himself."

3. "The homœopathic potencies are not nothings, but, indeed, as Hahnemann has perfectly recognized, not weakenings of the efficaciousness, but an augmentation, therefore, a veritable potentiation."

4. "*The main pillar upon which Homœopathy rests, is the Principle of Potentiation: with it it stands, without it it falls.*"

